# Gut Microbiota–Gut Metabolites and *Clostridioides difficile* Infection: Approaching Sustainable Solutions for Therapy

**DOI:** 10.3390/metabo14010074

**Published:** 2024-01-22

**Authors:** Bijay Gurung, Maranda Stricklin, Shaohua Wang

**Affiliations:** 1 Department of Biomedical Sciences, Ohio University Heritage College of Osteopathic Medicine, Ohio University, Athens, OH 45701, USA; bg959221@ohio.edu (B.G.); ms019121@ohio.edu (M.S.); 2 Infectious and Tropical Disease Institute, Ohio University, Athens, OH 45701, USA; 3 Interdisciplinary Molecular and Cellular Biology Program, Ohio University, Athens, OH 45701, USA

**Keywords:** *Clostridioides difficile*, gut microbiota, gut metabolites, probiotics, bile acids, SCFAs, gut microbiota modulator

## Abstract

*Clostridioides difficile* (*C. difficile*) infection (CDI) is the most common hospital-acquired infection. With the combination of a high rate of antibiotic resistance and recurrence, it has proven to be a debilitating public health threat. Current treatments for CDI include antibiotics and fecal microbiota transplantation, which contribute to recurrent CDIs and potential risks. Therefore, there is an ongoing need to develop new preventative treatment strategies for CDI. Notably, gut microbiota dysbiosis is the primary risk factor for CDI and provides a promising target for developing novel CDI therapy approaches. Along with gut microbiota dysbiosis, a reduction in important gut metabolites like secondary bile acids and short-chain fatty acids (SCFAs) were also seen in patients suffering from CDI. In this review study, we investigated the roles and mechanisms of gut microbiota and gut microbiota-derived gut metabolites, especially secondary bile acids and SCFAs in CDI pathogenesis. Moreover, specific signatures of gut microbiota and gut metabolites, as well as different factors that can modulate the gut microbiota, were also discussed, indicating that gut microbiota modulators like probiotics and prebiotics can be a potential therapeutic strategy for CDI as they can help restore gut microbiota and produce gut metabolites necessary for a healthy gut. The understanding of the associations between gut microbiota–gut metabolites and CDI will allow for developing precise and sustainable approaches, distinct from antibiotics and fecal transplant, for mitigating CDI and other gut microbiota dysbiosis-related diseases.

## 1. Introduction

*Clostridioides difficile* (*C. difficile*) is a Gram-positive, spore-forming anaerobic bacterium producing Toxins A and B, major virulence factors causing *C. difficile* infection (CDI). CDI is the leading cause of hospital-associated diarrhea, and can cause significant morbidity and mortality, along with colitis. CDI leads to more than 500,000 emergency visits and around 29,000 deaths each year in the United States alone, which has been attributed to cause an estimated loss of USD 6.3 billion [1]. After the initial infection, about 20–35% of patients develop recurrent CDI, and among them, roughly 40–60% will experience a second recurrence [2]. Vancomycin and fidaxomicin are the current antibiotics used to treat CDI. This treatment is becoming ineffective with the increasing instance of the antibiotic resistance of *C. difficile*, notably during the pandemic, due to the overuse of antibiotics [3]. CDI has been identified as an “urgent threat” by the Centers for Disease Control (CDC), and this emphasizes timely intervention and action to treat the disease and prevent recurrence [4]. 

The risk factors of CDI include age, obesity, hospitalization, antibiotic usage, and proton pump inhibition [5,6,7,8]. Antibiotic usage has been regarded as one of the most significant risk factors associated with CDI [5]. Prolonged or multiple antibiotic treatments can alter the composition of the gut microbiome and leads to dysbiosis, which enhances the possibility of CDI [6]. Similarly, in other cases like obesity, there is a decrease in the alpha diversity of the gut microbiota, which can enhance the pathogenesis of CDI [9,10]. Since one of the major factors related to the occurrence of CDI is gut microbiome dysbiosis, fecal microbiota transplantation (FMT) has been used as a treatment in recurrent CDI cases to improve the gut microbiome and prevent recurrent CDI [11,12]. However, due to the complex factors associated with the pathogenesis of CDI, the current antibiotic and FMT treatment strategies are ineffective for an optimal clinical outcome [13,14,15,16]. The current strategy against CDI may successfully kill vegetative cells with antibiotic treatment, but does not affect the spores, which can germinate and multiply, leading to recurrent CDI [6]. Although FMT has been a valuable strategy for preventing recurrent CDI, risk factors such as the transmission of pathogenic organisms complicate the FMT strategy, and this has lead the Food and Drug Administration (FDA) to alter the use of FMT [17]. 

Although the gut microbiota is an essential aspect of CDI, the treatment strategy often overlooks the role of the gut microbiota in the pathogenesis of CDI. The gut microbiota of healthy individuals plays an integral role in providing resistance against the colonization of *C. difficile* by competition for resources, the production of bacteriocins, and secondary metabolites that inhibit the germination of *C. difficile* spores [11,15,18,19]. Inversely, there are a group of microbes that enhance the pathogenesis of CDI [16]. Therefore, studying the interactions of the gut microbiota, gut metabolites, and their influences on CDI is crucial to better understanding CDI pathogenesis and developing a sustainable therapeutic strategy. 

This review will explore and discuss the current research studies in understanding the gut microbiota signatures to predict the pathogenesis of CDI, the roles of different metabolites in CDI pathogenesis, various gut microbiota modulators, and potential therapies against CDI.

## 2. Gut Microbiota and CDI

Bacteroidetes and Firmicutes are the dominant phyla of gut microbiota. Gut microbiota also comprises species from other phyla such as Actinobacteria, Proteobacteria, Fusobacteria, and Verrucomicrobia [20]. The majority of bacteria in the gut from phylum Bacteroidetes are from *Bacteroides* and *Prevotella* genera [21]. *Firmicute*’s composition in the gut microbiota is diverse, encompassing genera including *Lactobacillus*, *Bacillus*, *Clostridium*, *Enterococcus*, *Ruminicoccus*, *Faecalibacterium*, and *Roseburia* [21]. The *Bifidobacterium* genus dominates the gut microbiota from the Actinobacteria phylum [21]. 

The gut microbiota is essential for general wellbeing. The benefits of normal gut microbiota include immune system modulation, pathogen inhibition, and the production of several beneficial metabolites [22]. Regarding CDI pathogenesis, the gut microbiota plays an important role. Several risk factors of CDI include old age, diet, hospitalization, antibiotics, and proton pump inhibitor treatment [20,23,24,25,26], which could alter the gut microbiota composition. *Bifidobacteria* species are dominant in infants and their proportion decreases with age [27]. Gut dysbiosis is a characteristic of patients suffering from CDI, where the alpha diversity of the microbiome is lower compared to healthy individuals [28,29]. This information regarding changes in the gut microbiome population should be integrated into the CDI diagnosis and pathogenesis for novel treatment development.

Several studies have shown that CDI accompanies a marked decrease in the diversity of gut microbiota populations [30,31,32,33]. One of the several mechanisms by which the decreased gut microbial diversity contributes to CDI pathogenesis is due to weakened colonization resistance against *C. difficile* [34,35]. CDI is a hospital-associated infection because of the use of antibiotics and proton pump inhibitors and the potential transfer of *C. difficile* spores [23,34,36]. Antibiotics and proton pump inhibitors modulate and reduce the diversity of the gut microbiota. The recovery of the gut microbial population after antibiotic treatment may take some time, depending on the individual [37]. A delicate balance of the gut microbial population is essential for the wellbeing of an individual in preventing and defending against CDI.

One of the significant problems associated with treating CDI is reoccurrence, which becomes more challenging to treat [36,38]. The current diagnosis test for CDI involves the enzyme immune assay (EIA) for Toxin A, or a more sensitive test involves the nucleic acid amplification test (NAAT), which detects the toxin genes [39,40]. However, there is a discrepancy in the results, with diagnostic tests being unable to provide accurate information regarding the severity of CDI [40]. CDI and gut microbiota are closely related, and therefore, information regarding the change in the gut microbiota can give insight into the role of different gut microbes in the pathogenesis of CDI. By studying the gut microbiota in patients having recurrent CDI with healthy individuals, CDI patients with no recurrent CDI can provide insightful information about the composition of the gut microbes and differences between the groups. This information can then be used to determine the microbial signatures to help identify the patients at risk of getting CDI and recurrent CDI. Several studies have been done to understand the changes in the gut microbiota composition of patients with CDI [30,31,32,33,41]. The proteobacteria phylum was increased in the patients with CDI, where *Enterobacteriaceae* was a significant family contributing to that increase [28,31,42]. Similarly, Firmicutes and Bacteroidetes, two of the dominant phyla, were found to be decreased in CDI, and *Lachnospiraceae* and *Ruminococcaceae* families from the Firmicutes and *Bacteroides* genera from Bacteroidetes were the major contributing factors to the reduced proportion [42]. Regarding the Actinobacteria phylum, its proportion was found to be decreased in CDI, with *Bifidobacterium* being a significant contributing genus [42].

The abundance of the specific genera of bacteria could provide information regarding the severity of the CDI (Table 1). A study by Vázquez-Cuesta et al. identified several microbial genera that can be used as biomarkers or signatures for predicting CDI [31]. Some of the genera they identified that could be used for the biomarkers of CDI were *Bacteroides*, *Proteus*, *Robinsoniella*, *Paraprevotella*, and *Eggerthella*, as well as *Veillonella*, *Fusobacterium*, *Enterococcus*, and *Lactobacillus* for recurrent CDI [31]. Similarly, another study found that patients with an increased abundance of *Bacteroides*, *Ruminococcaceae*, *Clostridiacea*, and *Faecalibacterium* had better responses to CDI treatments compared to the patients who had recurrent CDI, who had increased populations of *Veillonella*, *Enterobacteriaceae*, and *Parabacteroides* [42]. Several studies have shown that higher CDI severity is associated with an increased proportion of *Enterobacteriaceae* and *Enterococcus* and a decreased proportion of *Lachnospiraceae* and *Bacteroidaceae* [32,33,41,42]. 

As well as bacteria, fungi also make a significant contribution to the gut microbiota. Minimal studies have been done on understanding their roles in CDI. A study by Cao et al. [43] analyzed the fungal composition in CDI patients and compared it with non-CDI patients. Their research found that mycobiota diversity was comparatively lower in CDI patients compared to the non-CDI group, where phylum Ascomycota was significantly higher and Basidiomycota was lower in the CDI patients [43]. The ratio of Ascomycota to Basidiomycota could be used as a biomarker for CDI [43]. More studies are required in the mycobiota to understand its potential role in CDI, which could further strengthen the options for therapeutic strategies against CDI.

## 3. Gut Metabolites and CDI

Other than the importance of the gut microbiota in providing colonization resistance against *C. difficile*, several other aspects of the gut microbiome play a role in the wellbeing of an individual and, ultimately, the protection against *C. difficile* and other pathogens [26,44,45,46]. One such important aspect is gut microbiota-derived metabolites. Depending upon the phylum and genera of the bacteria, they are involved in producing a variety of metabolites that can either suppress or aid the pathogenesis of CDI [15,47,48].

Due to the strict anaerobic nature of *C. difficile*, spores of *C. difficile* are significant aspects of CDI pathogenesis and transmit *C. difficile* from one host to another [34]. Toxins A and B, secreted by the vegetative cells of *C. difficile* [49], are major players in the pathogenesis of the CDI. Once the spores enter the gut, they germinate to produce toxins, causing the disease. The normal gut microbiota provides colonization resistance and inhibits spore germination by producing gut metabolites such as secondary bile and short-chain fatty acids (Table 2) [14,26,35,50]. Some metabolites such as [51] bile acids and amino acids have been found to enhance the germination of the spores [52,53,54,55,56].

### 3.1. Bile Acids

Bile acids have been widely studied for their role in CDI pathogenesis [44,56,59,60,61]. The liver produces two types of primary bile acids: cholic acid (CA) and chenodeoxycholic acid (CDCA) [51,62]. Bile acid synthesis is regulated by the Farnesoid X receptor (FXR) [63]. These bile acids are modified via conjugation with glycine or taurine to form glycocholic acid (GCA)/glycocheodeoxycholic acid (GCDCA) or taurocholic acid (TCA)/taurochenodexoycholic acid (TCDA) before being released into the intestines [51,62]. Bile acids are primarily involved in the emulsification and absorption of fats. The bile acids undergo enterohepatic recirculation, where conjugated and unconjugated bile acids are passively reabsorbed in the small intestine and colon. In contrast, in the distal ileum, the active reabsorption of bile acid takes place. Around 95% of the bile acids are reabsorbed during enterohepatic recirculation [64]. Several gut microbes can bio-transform the remaining 5% of the bile acids [51,65]. Secondary bile acid formation is the common biotransformation of the primary bile acids, which involves enzymes like bile salt hydrolase and 7-αdehydroxylating enzymes [44,62,65]. Bile salt hydrolase is an essential enzyme responsible for deconjugating the primary bile acids, which is responsible for the hydrolysis of the amide bonds of the conjugated bile acids [62]. The deconjugation process is an essential step because it prepares the bile acids to be acted upon by 7-αdehydroxylating enzymes, which only work on the deconjugated bile acids and generate secondary bile acids deoxycholic acid (DCA) and lithocholic acid (LCA) from CA and CDCA, respectively [51]. The responsible enzymes are encoded by the bile acid-inducible (bai) operon, present on very few gut microbes, mostly from the *Clostridium* genera [65,66]. Primary bile acids, CA, and TCA play a significant role in *C. difficile* spore germination, whereas chenodeoxycholic acid derivates can repress germination [52,56]. Besides inhibiting spore germinations, CDCA derivates have also been found to inhibit the growth of vegetative cells [25,59,67]. In the gut, bacterial genera like *Bacteroides*, *Blautia*, *Eubacterium*, *Clostridium*, and *Roseburia* were responsible for encoding bile salt hydrolase [68], whereas 7-αdehydroxylating enzymes were produced by a few groups of gut microbes belonging to *Ruminococcaceae*, *Lachnospiraceae*, and *Peptostreptococcaceae* [69]. 

Several studies have been performed to understand the role of secondary bile acids in *C. difficile* inhibition [44,55,59,67]. Binding to Toxin B by secondary bile acids was shown in an experiment conducted by Tam et al., where it led to conformational changes in Toxin B, making it unable to bind to the cell surface receptors [67]. Similarly, another possible mechanism of secondary bile acids on *C. difficile* inhibition is via the activation of an anti-inflammatory response, for which a study showed that the administration of ursodiol (a secondary bile acid) reduced the expression of inflammatory response factors like the interleukin 1 receptor and toll-like receptors [59]. Similarly, besides its direct role in *C. difficile* inhibition, it could regulate the production of antibiotics from the gut microbes. A study by Kang et al. showed that *Clostridium scindens* and *Clostridium sordellii* were involved in the production of tryptophan-based antibiotics, which showed an inhibitory effect against *C. difficile*; furthermore, a secondary bile acid concentration regulated the antibiotic activity [44]. Similarly, a study by Hazelton et al. showed that mice fed with a high-fat diet experienced an increase in the severity of CDI due to the increased production of primary bile acids to digest fats, which led to a decrease in the diversity of the gut microbiota via the reduction of secondary bile acids [70]. A study by Jose et al. used Obeticholic acid, an Farnesoid X receptors (FXR) agonist, to reduce the primary bile acid synthesis and decrease the severity of CDI in the high-fat-diet obese mice [10]. A decrease in the abundance of these genera of microbial populations due to antibiotics, proton pump inhibitors, and other comorbidities can lead to decreased secondary bile acid production, which aids in *C. difficile* spore germination to cause CDI. 

### 3.2. Short-Chain Fatty Acids (SCFAs)

Short-chain fatty acids (SCFA) are another critical group of metabolites that play significant roles in CDI [48,71,72]. Some examples of SCFAs are butyrate, acetate, succinate, and propionate, which are one-to-six carbon units long [48]. Gut microbes produce SCFAs via the fermentation of non-digested carbohydrates and from branched-chain amino acids [73]. So, the concentration of the SCFAs depends on factors like the composition of SCFA-producing gut microbes and the dietary intake. The difference in the composition of gut microbes can lead to differences in the composition of the SCFAs produced [74]. Tsukuda et al. found that gut microbes representing the order Clostridiales were involved in producing butyrate and propionate, whereas gut microbes from Enterobacterales produced succinate. Bacteria from the *Bifidobacterium*, *Blautia*, and *Roseburia* genera are involved in acetate production [74].

Regarding the role of SCFAs in CDI pathogenesis, a decrease in the proportion of SCFA-producing bacteria and a simultaneous reduction in SCFAs has been seen in patients suffering from CDI [48]. Several studies have shown that SCFAs like butyrate, propionate, and acetate provide resistance against *C. difficile* [19,48,58,75,76]. SCFAs have benefits for human health. In the gut, they maintain homeostasis and improve intestinal integrity. SCFAs can benefit the host by involving different signaling pathways, leading to improved gut health. SCFAs are shown to bind receptors like free fatty acid receptors 3 and 2 (FFAR3, FFAR2), G-protein coupled receptors 109a (GPR109a), and olfactory receptor-78 (Olfr78) [72]. These receptors are involved in the activation of several cellular responses like immune regulation and metabolic homeostasis [46].

The disruption of the gut barrier is one of the clinical manifestations of CDI, a weakened barrier can exacerbate the CDI as toxins (tcdA and tcdB) are released from multiplying *C. difficile* cells across the intestinal barrier, which can elicit inflammatory responses and worsening of the symptoms [34,49]. One of the mechanisms that aid in CDI inhibition is the strengthening of the intestinal barrier by SCFAs. Among the SCFAs, the inhibitory effects of butyrate on CDI pathogenesis have been widely studied [19,46,58,77,78]. In normal conditions, butyrate plays an essential role in the proliferation of colon epithelial cells, modulating the immune response, strengthening the intestinal barrier, and regulating the gut microbiota [19,71]. A study by Wang et al. showed that butyrate could inhibit CDI via the improvement of intestinal structural integrity, the regulation of the bile acid metabolism, and the regulation of the anti-inflammatory response. Butyrate enhanced the intestinal integrity by increasing the expression of tight junction proteins, Claudin-1 and Occludin [58]. Similarly, acetate, which can bind FFAR2, is involved in activating a pathway that recruits neutrophils and increases the secretion of interleukin 1β (IL-1β), which can prevent the translocation of bacteria [79]. Similarly, Toxin A produced by *C. difficile* inhibits microtubule formation by activating histone deacetylase-6 (HDAC-6), leading to mucosal damage. Acetate can hinder the process by binding to HDAC-6 and maintaining structural integrity [80].

## 4. Gut Microbiota Modulators

As CDI is associated with changes in the composition of the gut microbiota, studying factors that can modulate the gut microbiota will provide valuable information necessary for combating CDI.

### 4.1. Antibiotics

Regarding the CDI, the significant factors negatively modulating the gut microbiome would be drugs like antibiotics and proton pump inhibitors used to treat different diseased conditions [7,35,37]. Some CDI-associated antibiotics are clindamycin, monobactams, penicillin, carbapenems, cephalosporins, and fluoroquinolones, with clindamycin having the highest correlation with CDI [8]. In a study by Buffie et al., clindamycin treatment significantly reduced the gut microbial diversity and markedly increased bacterial populations, with a minor proportion in the normal gut microbiota, in the mice and made them prone to CDI [81]. An increase in *Enterobacteriaceae* populations with clindamycin treatment was correlated with the onset of CDI; in contrast, a decrease in CDI pathogenesis was associated with the reduction of the *Enterobacteriaceae* population and restoration of bacterial populations from *Bacteroides* and *Porphyromonadaceae* [82].

### 4.2. Proton Pump Inhibitors (PPIs)

Like antibiotics, proton pump inhibitors (PPIs) can modulate the gut microbiota and prompt CDI pathogenesis [7,83,84]. PPIs are generally used for treating diseased conditions like stomach ulcers and chronic acid reflux, which act by decreasing the acid production in the stomach. Suppressing the acid production in the gut can alter the pH, i.e., reduce the stomach’s acidity [85]. Stomach acidity acts as a defense by inhibiting several bacteria; however, a decrease in acidity can lead to the increased survivability of the higher number of bacteria [7]. A study by Imhann et al. showed that PPI usage increased the proportion of oral microbiota in the gut [7]. In addition to this modulation, the PPIs used resulted in decreased alpha diversity accompanied by a decrease in the *Ruminococcaceae* and *Bifidobacteriaceae* families and an increased proportion of *Enterococcaceae*, *Lactobacillaceae*, *Veillonellaceae*, and *Enterobacteriaceae* [7].

### 4.3. Probiotics

Probiotics are live microorganisms known to have a beneficial effect on the body [86]. The modulation of the gut microbiota is among the beneficial effects of the probiotics on the host [87,88]. Some widely used probiotics are *Bifidobacterium*, *Lactobacillus*, and *Lacticasiebacillus* [87]. Many studies have shown that probiotics modulate gut microbiota composition [89,90,91]. Wu et al. showed that *Akkermansia muciniphila* protected against *C. difficile* via the modulation of the gut microbiota and the anti-inflammatory response in mice. The mice treated with the *Akkermansia muciniphila* group had a decreased abundance of *Enterobacteriaceae* and *Enterococcoaceae*, whose proportion is comparatively higher in CDI [92]. Probiotic usage can modulate the gut microbial composition by influencing processes like SCFA production [93,94,95]. A study by Moens et al. used a formulated aqueous solution of four probiotic strains to study their colonization and influence on SCFA production. Their study revealed that the probiotic strains could colonize and proliferate in the proximal and distal columns and increase lactate production [93]. The increased lactate production contributed to the modulation of gut microbiota by increasing the population of bacteria that can consume lactate and convert it into butyrate [93]. Another possible mechanism of gut microbiota modulation by probiotics can be related to its inhibitory effects against several pathogens. Probiotics can inhibit pathogens by modulating the gut pH and competing for nutrients. Additionally, the production of antimicrobial compounds can directly inhibit the pathogens, which can help modulate the gut microbiota further [46,91,96].

Although it has been studied as a gut microbiota modulator in many studies, the efficiency of probiotics in gut microbiota modulation is inconsistent in clinical studies, where several studies show no significant differences in the gut microbiota between the probiotic-treated individuals and the placebo group [97,98,99,100]. A study by Wolfe et al. explored the role of probiotics in modulating the gut microbiota. Probiotic strains from the *Lactobacillus* and *Bifidobacterium* genera given to the patients with CDI reduced diarrheal symptoms, and no significant differences in the community diversity were seen [97]. Although there were no significant differences in the gut microbiota community diversity, apparent changes at the family level were seen; *Verrucomicrobiaceae* and *Bacteroides* were comparatively lower in the probiotic group [97]. Both treatment groups had an increased proportion of *Lachnospiraceae*, especially the *Ruminococcus* genera. However, the increment was slower in the non-probiotic-treated patients, which could be one of the reasons for alleviating diarrheal symptoms in the probiotic-treated patients, as *Ruminococcus* has been shown to inhibit *C. difficile* [97]. 

### 4.4. Prebiotics

Prebiotics, a substrate orthogonally utilized by the beneficial gut microbes [86], are another critical factor that can modulate the gut microbiota. Compounds from fructooligosaccharides and galactooligosaccharides are two major prebiotic groups that positively benefit human health [101,102]. These prebiotics are present in low quantities in the regular food we consume and are degraded by the gut microbes to generate SCFAs [101]. Gut microbes utilize prebiotics as an energy source, which can aid in modulating the gut microbiota [88,89,101]. For example, inulin, a widely studied prebiotic, can modulate the gut microbiome by stimulating the growth of *Bifidobacteria* and *Lactobacillus* [103,104]. One of the mechanisms by which prebiotics can modulate the gut microbiota is through the SCFA-mediated regulation of G-protein-coupled receptors. The increased production of the SCFAs due to prebiotic supplementation can activate FFAR2 expression, which can be involved in repressing the growth of gut microbes from *Prevotellaceae* and increasing the population of *Bifidobacterium* [105]. Similarly, prebiotics can effectively modulate the gut microbiota by inhibiting the adhesion of the pathogenic bacteria in gut epithelial cells. A study by Ribeiro et al. showed that prebiotics derived from olive pomace decreased the adhesion of the pathogenic bacteria *Listeria monocytogenes* and *Bacillus cereus* to mucin. Along with that, the use of prebiotics promoted SCFA production [106].

## 5. Therapeutical Strategies against CDI

### 5.1. Antibiotic Therapy

Antibiotics such as metronidazole, vancomycin, and fidaxomicin are first-line drugs for treating CDI [40]. The choice of antibiotics can be made based on the severity of the CDI, where metronidazole used to be recommended as an initial therapy, and vancomycin was administered to patients showing higher disease severity [107]. However, metronidazole is no longer recommended as a treatment for CDI due to the development of resistant *C. difficile* and the lower effectiveness in clearing *C. difficile* [108]. Compared to vancomycin, fidaxomicin is a narrow-spectrum antibiotic, which can have a reduced impact on the gut microbiota. The mode of action of fidaxomicin involves the inhibition of bacterial transcription, which has been shown to inhibit RNA polymerase activity by binding to the sigma subunit [109]. An in vitro study regarding fidaxomicin’s role as an antimicrobial agent revealed that it is a promising agent for the inhibition of Gram-positive bacteria, with the highest activity shown against *C. difficile*, and showed some degree of inhibition against other Gram-positive bacteria like enterococci and staphylococci [110]. In contrast, it had significantly lower activity against the Gram-negative aerobes and anaerobes [110]. One of the risk factors associated with vancomycin therapy is the possibility of recurrent CDI, as vancomycin, along with its role in inhibiting *C. difficile*, also disrupts the normal gut microbiota, leading to increased chances of recurrent CDI [35,36,111]. A study by Cornely et al. comparing the effectiveness of vancomycin and fidaxomicin treatment against CDI and recurrent CDI showed that fidaxomicin and vancomycin had similar responses in alleviating the CDI symptoms. However, fidaxomicin had a lower recurrence rate than vancomycin, i.e., 20% vs. 35% for fidaxomicin and vancomycin, respectively [112]. Although the development of fidaxomicin has led to more specific treatment strategies, several factors, including the higher cost of fidaxomicin treatment, outweigh its potential benefits [113].

### 5.2. Fecal Microbiota Transplantation (FMT)

Another widely used treatment strategy for CDI is fecal microbiota transplantation (FMT). FMT treatment strategy focuses on reducing the risk of CDI via the restoration of normal gut microbiota [114,115]. The treatment strategy involves reintroducing the gut microbiota in the CDI patients using fecal samples from a healthy individual [11]. FMT can provide resistance against *C. difficile* by improving colonization resistance due to the restoration of the normal microbiota, promoting the production of secondary bile acids and SCFAs, and strengthening the intestinal barrier via inducing the production of tight junctions and mucin [47,116]. FMT is generally used in the second recurrent CDI, where antibiotic therapy is ineffective [38]. Although several studies have shown the effectiveness of FMT in treating CDI, there have been several safety issues recently due to the lack of a defined standard protocol [117]. One of the risk factors associated with FMT is the transmission of pathogenic bacteria. DeFilipp et al. reported the bacteremia resulting from the extended-spectrum beta-lactamase (ESBL)-producing *E. coli* in FMT-transplanted patients, which resulted in the death of one patient [118]. This incident highlights the importance of screening the fecal samples for disease before using them for FMT, and even with screening, several risk factors can be associated with FMT [117]. As gut microbes are responsible for several physiological and biochemical processes, the impacts on these aspects of the host need to be studied more closely to understand FMT.

With the safety issues related to the FMT, and chances of recurrent infection in antibiotic-treated therapy, there is an urgent need for a more sustainable therapeutic strategy against CDI. Several other treatment strategies are being developed against CDI. One of the potential therapeutic strategies against CDI is the use of antibodies against *C. difficile* toxins. A study by Wilcox et al. studied the possible effects of actoxumab and bezlotoxumab for preventing recurrent CDI, which are antibodies against Toxins A and B, respectively. From their study, the usage of bezlotoxumab in the patients receiving antibiotic treatment had significantly lower recurrent infections compared to the control group, whereas actoxumab did not show any significant differences [119]. 

### 5.3. Phage Therapy

Another therapeutic strategy against CDI is bacteriophage therapy. Phage therapy could be an efficient and effective therapeutic strategy due the high specificity of bacteriophages and their inability to affect the normal gut microbiota [120]. A study by Nale et al. used seven different bacteriophages (six myoviruses and one siphoviruses) to study their inhibitory action against *C. difficile*. When used in combination, the bacteriophages were able to lyse 18 out of the 21 ribotypes of *C. difficile* [121]. However, the lack of strict lytic phages for *C. difficile* has created an obstacle for further studies in the phage therapy against *C. difficile* because the usage of the temperate phage can be associated with risk factors like the transfer and integration of viral DNA in *C. difficle* [121].

### 5.4. Probiotics as a Potential Therapy against CDI

Probiotics can be used as a potential therapeutic strategy for treating CDI. As mentioned before, probiotics can have several benefits to the host’s health via the modulation of the gut microbiota, pathogen inhibition, regulation, and the production of the important metabolites [96]. Several studies have been done to understand the potential use of probiotics in treating CDI. More extensive studies are needed to better understand the probiotics’ role in CDI prevention due to inconsistent results [98,99,100,122]. A study by Allen et al. showed that probiotic usage did not have any significant benefit in the prevention of CDI [99]. In addition, another study by Heil et al. also showed similar results, whereas a multi-year study showed no significant difference when high-CDI-risk patients were given probiotic supplements [100]. Several studies have shown the benefits of probiotics in treating CDI [122,123,124]. A study by Hudson et al. showed that patients receiving probiotic supplements and antibiotics had a lower incidence of CDI-associated diarrhea than those who did not receive the probiotic supplements [122]. Similarly, another study showed that using probiotics after the antibiotic treatment significantly reduced the incidence of CDI by 50% [123]. There are differences in the results, which might be due to the differences in several factors like the probiotic strains used, the age of the patients, dietary influence, comorbidity, and the probiotic dose. Similarly, the lack of a standard protocol related to probiotic usage may also lead to variations in the results. So, the research on the role of probiotics warrants further investigation if it is to be used for treating CDI. 

As CDI has been declared an urgent threat, there is a need for the development of new therapeutic strategies. One of the potential strategies that could be studied in the future is the development of specific probiotic mixtures based upon their functions and role and combining them with the prebiotics (a combination of probiotics and prebiotics are also known as synbiotic) against the CDI [104]. The dysbiosis of the normal microflora and drastic changes in gut metabolites like bile acids and SCFAs are characteristic features of CDI. Based on the information from the gut metabolites discussed above, we can see that the gut microbiota plays a vital role in the maintenance of a healthy gut via regulating different physiochemical processes and strengthening the gut epithelial tissues. Prebiotics play a vital role as a gut microbiota modulator, so the development of a probiotics mixture that has a specific role, for example, a combination of bile acids transforming probiotics and a SCFAs chain generating a probiotics mix, can help restore the gut metabolites, which can inhibit the *C. difficile* as well. Additionally, further research about different gut metabolites and small molecules produced by normal gut microbiota could be explored to find new compounds that can have higher specificity to *C. difficile* and a lower impact on the host gut microbiota.

Similarly, along with the need for the development of treatment strategies, there needs to be improvement in the diagnostic strategies as well. The diagnosis of pathogenesis is an important aspect needed for the creation of an effective therapeutic strategy. From Table 1, there are differences in the gut microbiota signatures between patients suffering from CDI, healthy individuals, and in asymptomatic carriers. Differences in the gut microbiota signatures could be used as a diagnostic test, which can help in developing an effective therapeutic strategy, based on which we could differentiate individuals that have a higher risk of CDI or recurrent CDI, and develop personalized therapeutic strategies for prevention. The incorporation of the other biomarkers and signatures like gut microbiome profiles, bile acid concentrations, SCFAs compositions, and other host-derived markers could improve the diagnosis of CDI, which can increase the efficiency of the therapeutic strategy. Mechanisms under the roles of gut microbiota in CDI have not been completely clarified. Further studies in understanding specific gut microbiota signatures and factors and mechanisms associated with the changes in the gut microbiota will be helpful to develop gut microbiota signatures as potential diagnostic tools and targets for CDI therapy. Similarly, gut metabolites were also found to play an important role in CDI pathogenesis (Table 2) and the wellbeing of an individual. Further research about those gut metabolites and CDI pathogenesis could provide better understanding about their mechanisms and can inform potential therapeutics.

## 6. Conclusions

**Figure 1:**
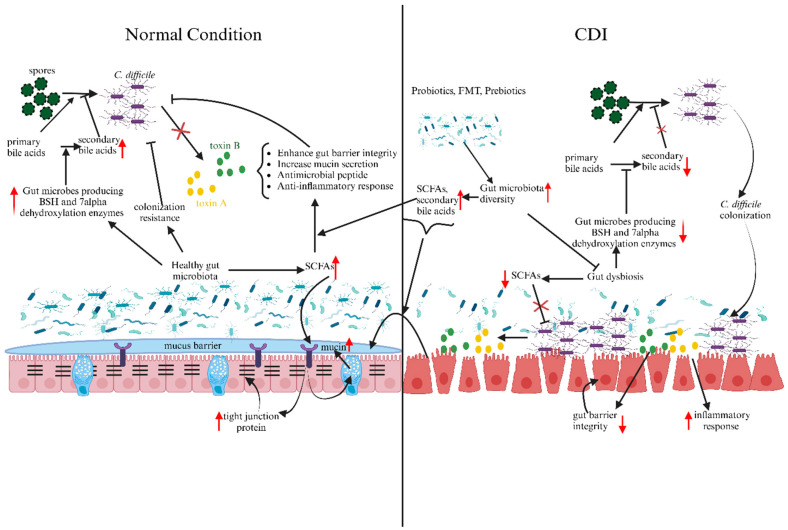
Differences in the gut environment between normal condition (**left**) and CDI (**right**). Gut dysbiosis leads to a marked decrease in gut microbiota diversity, which leads to a decrease in production of beneficial metabolites like SCFAs and secondary bile acids. A reduction in gut microbiota diversity, secondary bile acids, and SCFAs can enhance CDI pathogenesis, which can lead to increased disruption of gut barrier integrity and inflammatory response. The use of therapeutic strategies such as probiotics, FMT, and prebiotics can increase the gut microbiota diversity and alleviate gut dysbiosis. Moreover, it can increase the production of SCFAs and secondary bile acids, which can improve the resistance against CDI via improving gut barrier, anti-inflammatory response, and direct inhibition of *C. difficile*. Red arrows indicates either increase (upward arrow) or decrease (downward arrow) of gut microbial diversity, metabolites, gut barrier integrity and inflammatory responses. Figure created with BioRender.com.

The advent of new technologies has been remarkable for microbiome research, where it has led to a deeper understanding of the gut microbiota and its influence on the hosts. From the findings discussed above, the gut microbiota plays an important role in CDI pathogenesis. In a healthy individual, a normal gut microbiota plays an important role by inhibiting *C. difficile* via competition for nutrients, improving the gut barrier, gut metabolites, and antimicrobial compounds. However, the disruption of the gut microbiota by several factors can reduce the gut microbiota diversity, which can increase the potential for CDI from a decreased gut microbiota diversity that is unable to provide colonization resistance and gut metabolites necessary for *C. difficile* inhibition (Figure 1). Therapeutic strategies such as antibiotic and FMT are commonly used therapeutic strategies for CDI and have risk factors, such as recurrent CDI caused by the antibiotic used to treat CDI further disrupting the gut microbiota, and the transmission of pathogens and lack of standardized protocol from FMT. These risks further hamper the treatment of CDI. Similarly, in the diagnostic testing of CDI, despite the major role of the gut microbiota, there is no inclusion of the gut microbiota signature. The incorporation of gut microbiota signatures, along with the changes in the gut metabolites, can provide necessary information for the correct diagnosis of CDI, thereby enhancing the effectiveness of the therapeutic strategy.

The findings presented in this review highlight the importance of different factors in CDI pathogenesis such as bile acids, SCFAs, probiotics, and prebiotics. These factors could be further studied to understand their role in CDI prevention and could be used as a therapeutic strategy. Bile acids can be bio-transformed by specific gut microbiota to generate a secondary bile acid, which has been shown to inhibit the *C. difficile*. Future research regarding the bile acids’ mechanism in inhibiting *C. difficile* could help identify a specific molecule that may inhibit *C. difficile*. Similarly, there are several benefits associated with SCFAs for hosts and for prevention against CDI. The complex nature of CDI pathogenesis makes therapeutic strategies like antibiotics treatment ineffective. However, further research to identify and develop narrow spectrum antibiotics like fidaxomicin, specifically targeting *C. difficile*, could potentially provide effective therapeutic strategies in future. Moreover, gut microbiota dysbiosis is a major factor associated with CDI pathogenesis. Therapeutic strategies focusing on gut microbiota restoration can be another form of effective therapeutic strategy for treating CDI. Although FMT has already been used for treating CDI, the protocols need to be standardized to ensure that fecal samples are handled and screened extensively to reduce chances of the transmission of pathogenic organisms. So, alternative to FMT, probiotics along with prebiotics could be an effective therapeutic strategy for CDI in future. For example, combining specific groups of probiotics that can utilize prebiotics to form SCFAs along with group of probiotics involved in bile acid transformation could help in preventing CDI by improving gut health and providing colonization resistance against CDI. Therefore, future studies involved in developing a probiotic and prebiotic mix that can improve gut microbiota and prompt the production of secondary bile acids and SCFAs could be one of the sustainable approaches to alleviate CDI.

## Figures and Tables

**Table 1:** Gut microbiota signatures of CDI based on differences between healthy, asymptomatic carrier, CDI, and recurrent CDI patients.

Study Group	Result	References
CDI patients, recurrent CDI patients, non-*C. difficile* diarrhea patients, asymptomatic *C. difficile* patients, and control	Decrease in alpha diversity, with several genera like *Parabacteroides*, *Faecalicoccus*, and *Clostridium cluster XVIII* as potential biomarkers for colonization. For CDI, potential biomarkers included bacteria genera *Batceroides*, *Proteus*, *Paraprevotella*, and *Eggerthella*. For the recurrent CDI, *Veillonella*, *Enterococcus*, *Lactobacillus*, *Clostridium* cluster XIVa, etc., were potential biomarkers.	[31]
CDI patients, Asymptomatic carriers, non-CDI diarrhea, and Control	Lower diversity of the gut microbiome. Increased variation of immune markers between the study group. Several bacterial groups were identified as a potential influencer of CDI, like *Klebsiella*, *Streptococcus*, and *Veillonella*.	[30]
Mice were separated into groups based on human fecal samples used to inoculate the mice	Mice showing lower clinical scores and comparatively healthy had a higher proportion of *Akkermansia*, *Anaerotignum*, *Blautia*, *Enterocloster*, etc. Mice showing higher clinical scores had a prevalence of bacterial community from *Bacteroides*, *Enterococcus*, and *Klebsiella*.	[32]
Patients having both inflammatory bowel disease (IBD) and CDI, patients with just IBD, healthy control	Variation in the gut microbial (bacterial and fungal) diversity was significantly different between study groups. Bacterial species like *Enterococcus faecium*, *Clostridium inoculum*, *Ruminococcus gnavus*, and fungus *Saccharomyces cerevisiae* were found in high proportion in IBD-CDI patients.	[33]
Patients with primary CDI are further divided into two groups based on recurrent CDI	Calprotectin level combined with the gut microbiome composition provided better insight into the severity of the CDI. In patients with recurrent CDI, calprotectin level was higher, and it was accompanied by an increased proportion of Fusobacterium and a decreased proportion of *Ruminococcus*, *Prevotella*, and *Collinsella*	[41]

**Table 2:** Gut microbiota-derived gut metabolites and their roles in CDI pathogenesis.

Gut Metabolite	Result	References
Bile acids	*C. difficile* was involved in the increased flux of primary bile acids in the gut, enhancing spore germination and pathogenesis.	[56]
Secondary bile acids	In children who have ulcerative colitis and CDI, secondary bile acids like lithocholic acids in the fecal sample were significantly lower, and there was a significant decrease in the genes encoding for enzymes responsible for bile acid transformations.	[50]
Secondary bile acids	Antibiotic-associated gut microbiome disruptions led to decreased secondary bile acid production and increased outgrowth of *C. difficile* in the intestine. Bacteria from Firmicutes phylum, *Lachnospiraceae*, and *Ruminococcaceae* were involved in secondary bile acid production and, ultimately, showed resistance to *C. difficile*.	[26]
Secondary bile acids and antibiotics	Along with the production of the secondary bile acids DCA and LCA, *Clostridium cinders* and *Clostridium sordellii* were found to produce tryptophan-based antibiotics, which, in concert with secondary bile acids, had an excellent inhibitory effect against *C. difficile*.	[44]
Butyrate (SCFA)	Sodium butyrate was involved in the anti-inflammatory response via activation of GPR109A, which is involved in anti-inflammatory response, and reduces the intestinal permeability and increased production of tight junctions and Mucin 2.	[19]
Valerate (SCFA)	Clindamycin treatment was found to reduce the valerate concentration in the gut. Regarding the role of valerate in *C. difficile* pathogenesis, both in vitro and in vivo studies showed inhibitory effects against *C. difficile*.	[57]
Butyrate (SCFA)	Butryate levels were found to be reduced in patients with CDI. Regarding its protective activity, several mechanisms involved were bile acid metabolism regulation, intestinal barrier strengthening, and gut microbiota modulation.	[58]

## Data Availability

Not applicable.
